# Interacting Abiotic Factors Affect Growth and Mycotoxin Production Profiles of *Alternaria* Section *Alternaria* Strains on Chickpea-Based Media

**DOI:** 10.3390/pathogens12040565

**Published:** 2023-04-06

**Authors:** Cindy J. Romero Donato, María J. Nichea, Eugenia Cendoya, Vanessa G. L. Zachetti, María L. Ramirez

**Affiliations:** Instituto de Investigación en Micología y Micotoxicología, (IMICO), CONICET-UNRC, Ruta 36 Km 601, Río Cuarto 5800, Córdoba, Argentina

**Keywords:** *Alternaria alternata*, *Alternaria arborescens*, *Alternaria mycotoxins*, ecophysiology

## Abstract

Chickpea is susceptible to fungal infection and mycotoxin contamination. Argentina exports most of its chickpea production; thus, its quality is of concern. The *Alternaria* fungal genus was found to be prevalent in chickpea samples from Argentina. The species within this genus are able to produce mycotoxins, such as alternariol (AOH), alternariol monomethyl ether (AME), and tenuazonic acid (TA). In this context, we evaluated the effect of water activity (0.99, 0.98, 0.96, 0.95, 0.94, 0.92, and 0.90 a_W_), temperature (4, 15, 25, and 30 °C), incubation time (7, 14, 21, and 28 days), and their interactions on mycelial growth and AOH, AME, and TA production on chickpea-based medium by two *A. alternata* strains and one *A. arborescens* strain isolated from chickpea in Argentina. Maximum growth rates were obtained at the highest a_W_ (0.99) and 25 °C, with growth decreasing as the a_W_ of the medium and the temperature were reduced. *A. arborescens* grew significantly faster than *A. alternata*. Mycotoxin production was affected by both variables (a_W_ and temperature), and the pattern obtained was dependent on the strains/species evaluated. In general, both *A. alternata* strains produced maximum amounts of AOH and AME at 30 °C and 0.99–0.98 a_W_, while for TA production, both strains behaved completely differently (maximum levels at 25 °C and 0.96 a_W_ for one strain and 30 °C and 0.98 a_W_ for the other). *A. arborescens* produced maximum amounts of the three toxins at 25 °C and 0.98 a_W_. Temperature and a_W_ conditions for mycotoxin production were slightly narrower than those for growth. Temperature and a_W_ conditions assayed are those found during chickpea grain development in the field, and also could be present during storage. This study provides useful data on the conditions representing a risk for contamination of chickpea by *Alternaria* toxins.

## 1. Introduction

Since ancient times, chickpea (*Cicer arietinum* L.) has been an important crop, particularly in Asia, although over the years, it has been adapted to many continents including the Americas. The genus *Cicer*, which includes the cultivated chickpea, belongs to the family Fabaceae, subfamily Papilionoideae. Like other pulses, chickpeas are well known for their soil fertility restoration value due to their ability to fix nitrogen, helping agricultural systems to be productive and sustainable. Furthermore, chickpeas are highly nutritious, having high protein, fiber, and fat contents, but a lower carbohydrate content than wheat. Chickpea plants also contain many bioactive compounds, such as phenolic acid and isoflavones [1]. 

Globally, soybeans lead legume production, followed by peanuts, dried beans, dry peas, and chickpeas, among others [2]. In recent years, the global demand for legumes has increased, and this trend is expected to continue in the future. The average consumption of legumes in the world is about 8 kg per capita per year [3], while that of Argentina reaches barely 800 g per capita per year [4]. Most of the Argentinean chickpea production is destined for export, with India, Pakistan, Turkey, Brazil, and Colombia being the main buyers of Argentinian chickpeas [5,6].

However, there are some problems that threaten chickpea permanence as a profitable crop in the region. One of these problems are fungal diseases, often associated with the presence of toxic secondary metabolites, such as mycotoxins. Mycotoxins cause significant economic losses due to their impact on human and animal health and the rejection or reduction of prices during marketing. More than 25 fungal pathogens are known to colonize this crop before and/or after harvest and greatly affect productivity, altering the vigour and longevity of the seeds [7]. The more common fungal genera isolated from chickpea around the world are *Aspergillus*, *Fusarium*, *Penicillium*, *Alternaria,* and *Rhizopus* [8].

Due to the lack of information in Argentina about the contamination of this legume with mycotoxigenic fungi and mycotoxins, our research group recently evaluated the occurrence of mycotoxins in 70 asymptomatic chickpea samples from the Córdoba province (Argentina). We observed a high frequency of contamination with species belonging to the genera *Aspergillus* (54%) and *Alternaria* (22%), followed by *Fusarium*, *Chaetomium*, *Penicillium,* and *Rhizhopus*. At the same time, the natural occurrence of the different mycotoxins was evaluated using a multitoxin methodology by liquid chromatography coupled to mass spectrometry (LC-MS/MS). As a result, contamination with *Alternaria* toxins, such as alternariol (AOH), was observed in all samples analyzed at levels ranging between 0.7 and 14.5 ng/g and alternariol monomethyl ether (AME) in 30% of samples at levels ranging between 1.4 and 2.3 ng/g. In addition, *Fusarium* mycotoxins, such as beauvericin, deoxynivalenol, and zearalenone, were also detected [9]. Furthermore, due to the high prevalence of *Alternaria,* a phylogenetic analysis of 32 *Alternaria* strains isolated from chickpeas was carried out in order to identify the species based on the combined sequences of the *tef1*, *gpd,* and *Alt a1* genes. All *Alternaria* strains clustered into the section *Alternaria* and were identified as *A. alternata* and *A. arborescens*. In addition, we determined the toxigenic profile of each strain and observed that most of them were able to coproduce AOH, AME, and TA [10]. These results suggest that, when consuming chickpeas, a potential risk for human health could exist, because this legume could present contamination with *Alternaria* and with its mycotoxins, which are not yet regulated in food.

*Alternaria alternata* is considered the most frequently isolated species within the genus and is perhaps the most cosmopolitan, isolated worldwide from both temperate and tropical climates. *Alternaria arborescens* is also a ubiquitous fungus that can be found in many types of plants and other substrates. They can grow on a wide variety of substrates, including dead plant material, food, soil, leather, and textiles and as saprophytes on the surface of roots, leaves, and seeds [11,12,13,14,15,16,17,18,19,20]. 

Currently, over 70 secondary metabolites produced by the *Alternaria* species are known. Exposure to *Alternaria* toxins has been associated with a wide variety of adverse health effects in humans and animals. The main *Alternaria* mycotoxins detected in food are AOH, AME, and TA, which have mutagenic, carcinogenic, and genotoxic effects [21,22]. TA may act as a mycotoxin and phytotoxin and has been reported to be more poisonous than AOH and AME in several animal species, such as mice, chickens, and dogs [23].

Fungal contamination, both in the field and during the storage of agricultural products, depends largely on ecophysiological factors, such as the water activity (a_W_), temperature, pH, fungal strain, and substrate where it grows with the first two factors being the most important, particularly for the genus *Alternaria* [24,25]. For this reason, knowledge about how the environmental factors influence growth and mycotoxin production can represent an important tool for the prediction, understanding, and prevention of mycotoxin contamination in food. TA, AME, and AOH production by the *Alternaria* species in relation to these factors has been studied in different substrates including soybean, grape, and tomato [26,27,28,29], focusing on *A. alternata* to the detriment of others species. At the moment, there is no information about *A. alternata* and *A. arborescens* studies being carried in chickpeas. As a result, research about the behavior of these species in chickpeas would provide useful information in order to predict their growth with the ensuing mycotoxin contamination.

The objective of the present study was to determine the effects of a_W_ and temperature on the growth and production of AOH, AME, and TA by *A. alternata* and *A. arborescens* on a synthetic chickpea media. As a result, we provide more information about the influence of environmental factors on the contamination of this pulse with *Alternaria* toxins

## 2. Materials and Methods

### 2.1. Fungal Strains

Three *Alternaria* section *Alternaria* strains (*A. alternata* RC-CR 767 and RC-CR 902; *A. arborecens* RC-CR 910), previously isolated from chickpeas in Argentina during the 2018 harvest season, were used. They were previously identified by morphological and molecular criteria [30,31,32,33,34]. For the molecular characterization, the 1-α elongation factor (*tef1*), *Alternaria* major allergen (*Alt-a1*), and glyceraldehyde-3-phosphate dehydrogenase (*gpd*) gene were partially sequenced indicating that these isolates belong to the section *Alternaria* and can be characterized as *A. alternata* and *A. arborescens*. The ability of the strains to produce AOH, AME, and TA was determined in ground rice–corn steep liquor medium and detected by high-performance liquid chromatography (HPLC) [10]. All the strains are preserved at the Universidad Nacional de Río Cuarto (UNRC) culture (RC) collection as spore suspensions in 15% glycerol frozen at −80 °C.

### 2.2. Media Preparation

Chickpeas harvested in Argentina were finely milled by using a Romer mill (Romer Labs Inc., Union, MO, USA). Mixtures of 2% (*w*/*v*) of milled chickpea in water were prepared, and 2% (*w*/*v*) agar (technical agar no. 2, Oxoid, Basingstoke, UK) was added. The a_W_ of the media was modified by adding mixtures of water/glycerol instead of water to obtain target a_W_ treatments of 0.99, 0.98, 0.96, 0.95, 0.94, 0.92, and 0.90 before autoclaving for 15 min at 121 °C [35]. The molten cooled media were vigorously mixed before pouring into 9 cm Petri plates (20 mL per plate), cooled, and stored at 4 °C in sealed bags prior to use. The a_W_ of representative samples of media was checked with an Aqualab Series 3 (Decagon devices, Inc., Pullman, WA, USA). In order to detect any significant deviation of the media a_W_ values, uninoculated control plates were prepared and measured at the end of the experiment. 

### 2.3. Inoculation, Incubation, and Growth Assessment

Chickpea-based media were centrally inoculated with a mycelial plug of 3 mm diameter and removed with a sterile punch from the active growth area of each *Alternaria* section *Alternaria* strain grown on the synthetic nutrient agar (SNA) at 25 °C for seven days. After inoculation, plates with an equal a_W_ were placed in polyethylene bags and incubated at 4, 15, 25, and 30 °C for a period of 28 days. 

A full factorial design with 3 replicates was applied. The factors analyzed were strains (3) × a_W_ levels (7) × temperature (4), and as a response, the growth rate was analyzed.

### 2.4. Mycotoxin Analysis

For mycotoxin extraction after 7, 14, 21, and 28 incubation days, three agar plugs (4 mm diameter) were cut with a hole punch from the edge of a colony from each Petri plate, weighed, and placed in a 4 mL amber vial. The extraction method used for the three toxins was the microscale extraction technique proposed by Smedsgaard [36] modified in three extraction steps suitable for the mycotoxin produced by species of the genus *Alternaria* [37].

AOH, AME, and TA were detected and quantified by HPLC using the equipment and settings described by Nichea et al. [10]. The recovery rate of the method was >80% when different amounts of *Alternaria* mycotoxins (AOH, AME, and TA) were added (range 0.1 to 10 µg/g). For the three toxins, the limit of detection (LOD; signal-to-noise ratio 3) was 0.01 µg/g, and the limit of quantification (LOQ) was established as three times the detection limit.

### 2.5. Statistical Analysis

The growth rates and mycotoxin concentrations (transformed to Log_10_) were evaluated by analysis of variance (ANOVA) using SigmaStat for Windows version 2.03 (SPSS Inc., Chicago, IL, USA). Statistical significance was judged at the level *p* ≤ 0.001. When the analysis was statistically significant, the post hoc Tukey’s multiple comparison procedure was used for separation of the means (LSD-Fisher *p* < 0.05). Surface response and contour map graphs were performed using Sigma Plot v.10.0 (Systat Software Inc., Hounslow, London, UK).

## 3. Results

### 3.1. Effect of a_W_ and Temperature on Growth Rates

**Figure 1:**
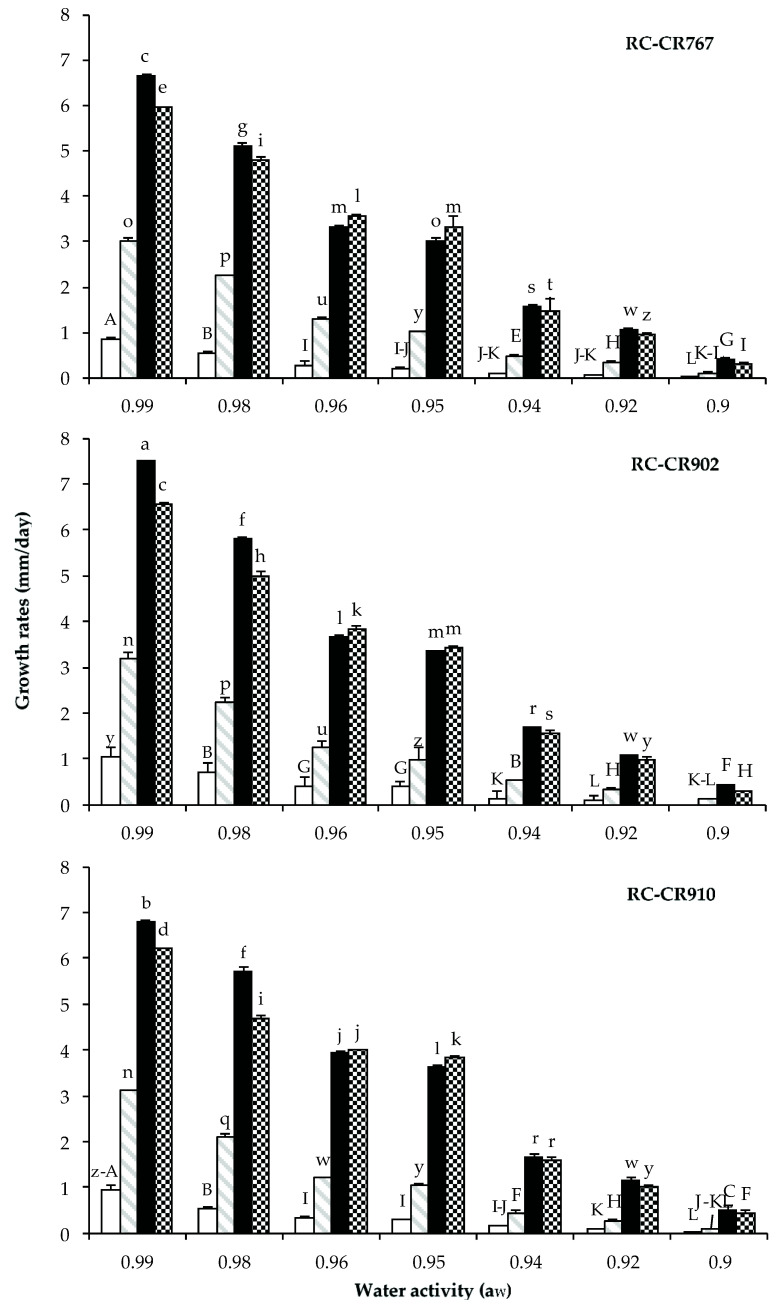
Effect of water activity (0.90–0.99) and temperature 4 °C (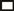), 15 °C (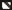), 25 °C (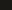), and 30 °C (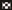) on growth rate (mm/day) of *Alternaria alternata* RC-CR767, *Alternaria alternata* RC-CR902, and *Alternaria arborecens* RC-CR910 in a chickpea-based media. Different letters indicate statistical differences (*p* < 0.05).

The results obtained for the growth rates of the three strains studied, under different a_W_ and temperature conditions, are presented in Figure 1. Both factors, a_W_ and temperature, influenced the mycelial growth rate of the three *Alternaria* section *Alternaria* species under study. The data show that *A. arborecens* RC-CR910 presented the highest growth rate followed by *A. alternata* RC-CR902 and finally by *A. alternata* RC-CR767. In general, the response of the growth rate to a_W_ and temperature was similar for both species. Maximum growth rates were observed at the highest a_W_ (0.99) evaluated at 25 °C, and they declined when the water availability in the media decreased. Regarding incubation temperature, higher growth rates were observed at 25 °C, decreasing in the following order: 30, 15, and 4 °C. Moreover, all of the strains under study were unable to grow at a_W_ levels ≤ 0.92 at 4 °C.

An analysis of variance of the effect of a single factor (a_W_, temperature, and strain) and its double and triple interactions revealed that all factors and their interactions had a significant influence on the growth rate. The factor that influenced the fungal growth the most was temperature followed by a_W_ and the temperature × a_W_ interaction (Table 1). A posteriori analysis showed that the strains, a_W_ levels, and the different temperatures studied were significantly different from each other (LSD-Fisher *p* < 0.05). 

**Figure 2:**
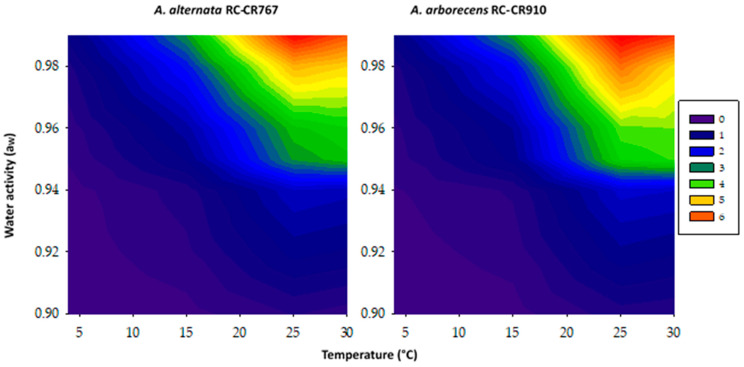
Two-dimensional contour maps of Alternaria alternata RC-CR767 and Alternaria arborecens RC-CR910 growth profiles in relation to temperature and water activity. Similar colors refer to similar growth rates (mm/day).

With the growth rate data obtained from the three strains evaluated (two *A. alternata* and one *A. arborescens*) under different growing conditions, contour maps were generated in an attempt to identify the optimum and marginal a_W_ and temperature conditions for growth (Figure 2). As both *A. alternata* behaved similarly, just one contour map is shown for this species. From these contour maps, the optimum conditions, regardless of the species, is around 20–30 °C at 0.99–0.95 a_W_. On the other hand, marginal conditions for growth are over a wider temperature range and limited to about 0.92–0.90 a_W_ levels.

### 3.2. Effects of a_W_, Temperature, and Incubation Time on Mycotoxin Production

**Figure 3:**
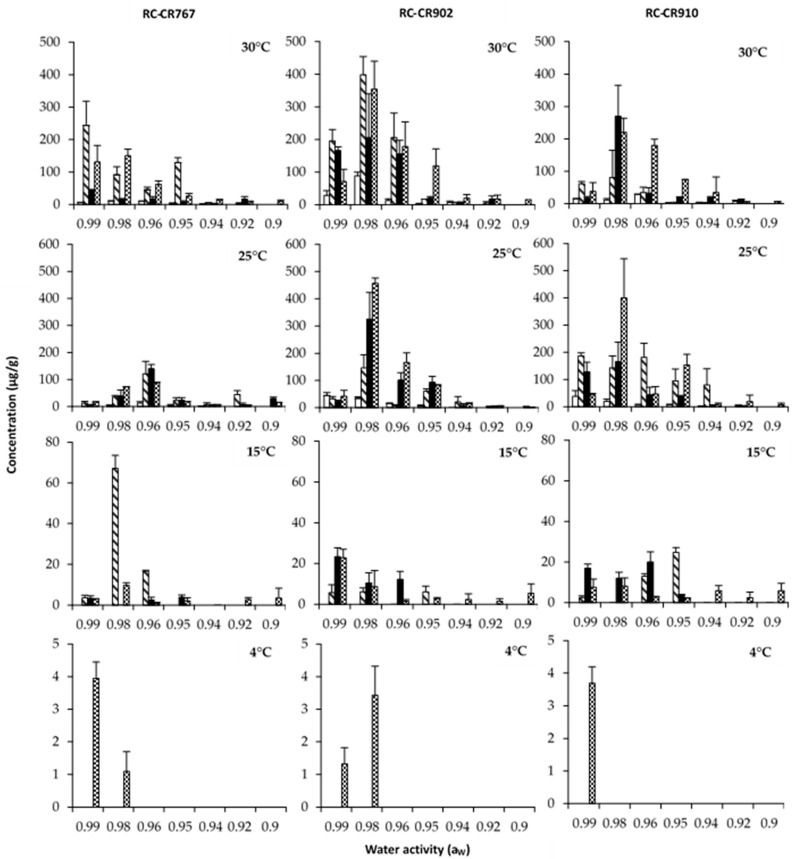
Altenariol concentrations (µg/g) produced by *Alternaria* section *Alternaria* strains: *Alternaria alternata* RC-CR767, *Alternaria alternata* RC-CR902, and *Alternaria arborecens* RC-CR910 in a chickpea-based media adjusted to different a_W_ levels and temperatures at different incubation times: 7 (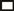), 14 (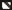), 21 (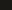), and 28 (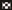) days.

**Figure 4:**
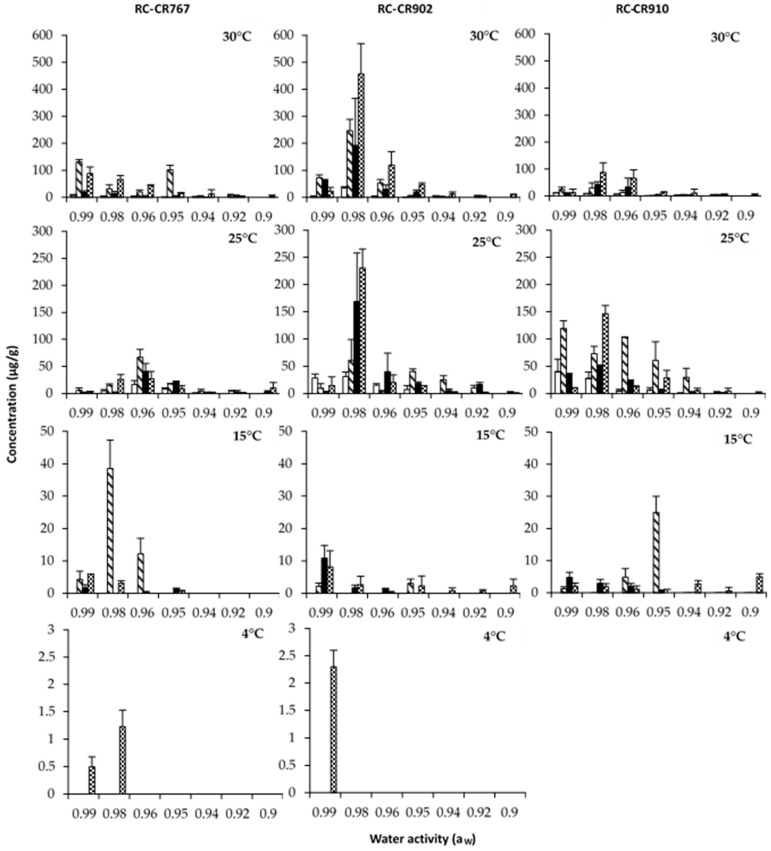
Altenariol monomethyl ether concentrations (µg/g) produced by *Alternaria* section *Alternaria* strains: *Alternaria alternata* RC-CR767, *Alternaria alternata* RC-CR902, and *Alternaria arborecens* RC-CR910 in a chickpea-based media adjusted to different a_W_ levels and temperatures at different incubation times: 7 (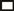), 14 (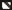), 21 (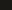), and 28 (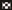).

**Figure 5:**
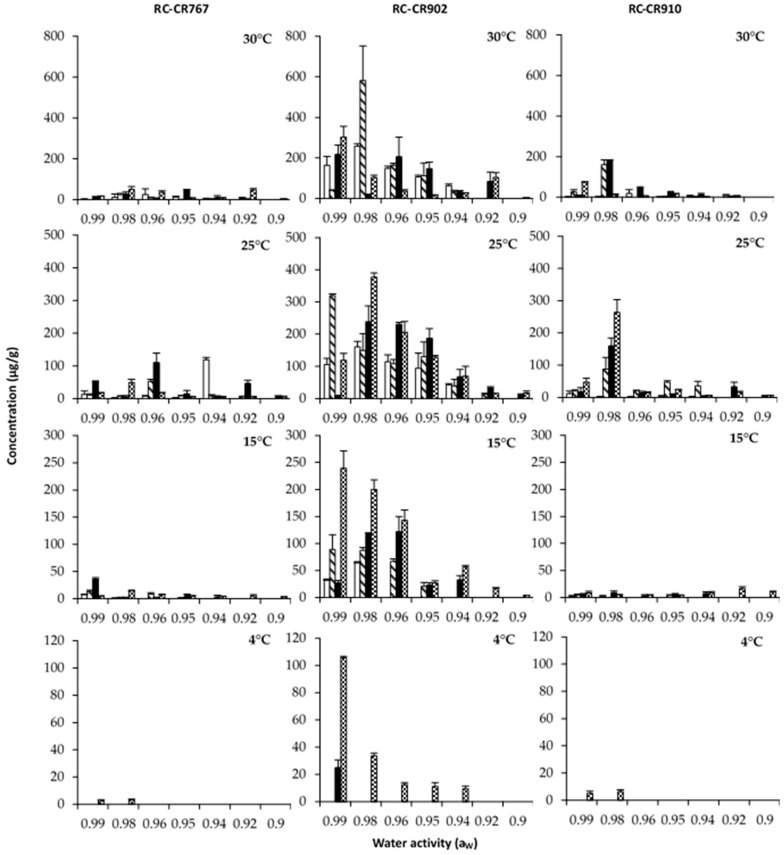
Tenuazonic acid (µg/g) produced by *Alternaria* section *Alternaria* strains: *Alternaria alternata* RC-CR767, *Alternaria alternata* RC-CR902, and *Alternaria arborecens* RC-CR910 in a chickpea-based media adjusted to different a_W_ levels and temperatures at different incubation times: 7 (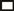), 14 (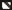), 21 (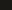), and 28 (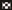).

Figure 3, Figure 4 and Figure 5 show the effects of a_W_, temperature, and incubation time on AOH, AME, and TA production by both *Alternaria* species grown in a chickpea-based media over 21 days. The results of this study show that the time course of production of the toxins evaluated varied with temperature and a_W_. Statistically, the effects of a_W_, temperature, and incubation time and their interactions were found to be significant (Table 2).

In general, the production of the three toxins increased with incubation time. The maximum production was reached at 14/21/28 days, depending on the toxin. Overall, the toxin concentrations produced by *A. alternata* RC-CR902 were higher than those produced by *A. alternata* RC-CR767 and *A. arborescens* RC-CR910 under almost all the tested conditions.

We will present the results separately for both *Alternaria* species, starting with both *A. alternata* strains.

AOH maximum production for *A. alternata* RC-RC902 was reached with temperatures of 25 and 30 °C at 0.98 a_W_ after 14 and 28 days of incubation (Figure 3). On the other hand, for the strain RC-CR767, the maximum level was obtained at 30 °C after 14 days of incubation at the highest a_W_ tested (0.99), followed by 25 °C at 0.96 a_W_ after 28 days of incubation. At 15 °C, a significant reduction in AOH production was observed for both strains. At the lowest temperature tested (4 °C), the production of this mycotoxin was almost inhibited; it was only detected, at very low levels, with 0.99 and 0.98 a_W_, after 28 days of incubation. 

The pattern of AME production was similar to those observed for AOH production by the two *A. alternata* strains (Figure 4). The highest AME production by the strain RC-CR767 was obtained at 0.99 a_W_ and 30 °C after 14 days of incubation, in comparison to the strain RC-CR902, which presented the highest level at 0.98 a_W_ and 30 °C after 28 days of incubation. At temperatures of 30 °C and 25 °C, the strain RC-CR902 showed high production levels at 0.98 a_W_, and mycotoxin concentration increased during the incubation time, while the strain RC-CR767 presented the most favorable conditions at 0.99 a_W_ at 30 °C and 0.96 a_W_ at 25 °C. At 15 °C, a significant reduction in AME production occurred for the two strains under study. At 4 °C, AME production was detected at trace levels of 0.99 and 0.98 a_W_. 

For TA production, a maximum concentration was obtained for *A. alternata* RC-CR 797 at 0.96 and 0.94 a_W_ and 25 °C after 21 and 14 days of incubation, respectively (Figure 5). RC-CR902 produced the maximum TA level at 0.98 a_W_ after 14 days of incubation at the highest temperature tested. In addition, for this strain at 15 °C, high levels of TA production were observed at 0.99, 0.98, and 0.96 a_W_ in comparison with the other *A. alternata* strain. At the lowest temperature tested (4 °C), a decrease in production levels was observed. However, these were still high compared to those observed for AOH and AME, especially by the strain RC-CR902 at ≥0.94 a_W_. 

In relation to *A. arborescens*, this strain reached the maximum production of AOH, AME, and TA at 0.98 a_W_ and 25 °C after 28 days of incubation. Considering the incubation temperature, the production of the three toxins was higher at 25 °C, decreasing in the following order: 30, 15, and 4 °C. At the lowest temperature tested (4 °C), no AME production was observed regardless of the a_W_, but traces of AOH and TA were detected at 0.99 and 0.98 a_W_ after 28 days of incubation.

**Figure 6:**
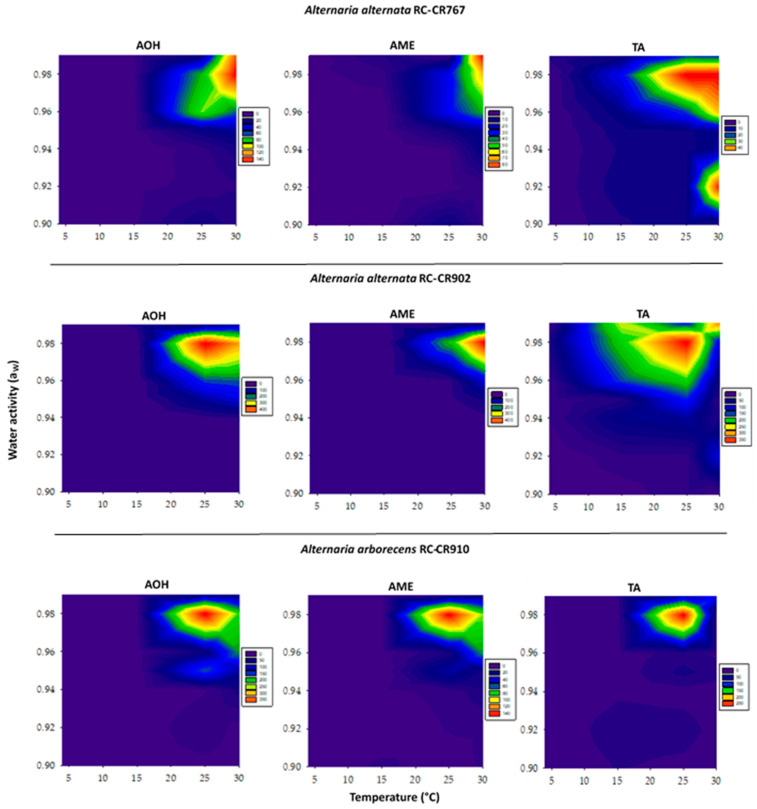
Two-dimensional contour maps of alternariol, altenariol monomethyl ether, and tenuazonic acid production by *Alternaria* section *Alternaria* strains: *Alternaria alternata* RC-CR767, *Alternaria alternata* RC-CR902, and *Alternaria arborecens* RC-CR910 in relation to temperature and water activity after 28 days of incubation. The numbers on the scale refer to toxin levels (µg/g).

The mycotoxin (AOH, AME, and TA) production data obtained were used to develop contour maps in order to identify the optimal and marginal conditions of a_W_ and temperature and the range of conditions for mycotoxin production after 28 days of incubation (Figure 6).

## 4. Discussion

To our knowledge, this study represents the first approach to understanding the interaction effect of a_W_ and temperature conditions on the growth and mycotoxin production by two *Alternaria* species belonging to section *Alternata* (*A. alternata* and *A. arborescens*) on chickpea-based media. It is important to remark that the strains used in the present study were previously isolated from chickpea produced in Argentina and identified using multigenic phylogenetic analyses [10]. All these details make the results obtained more reliable, because the taxonomy of the *Alternaria* genus is quite controversial. Both species *A. alternata* and *A. arborescens* share morphological features and are very difficult to be differentiated using traditional methods. This could be the reason why all the strains were classified in the past as *A. alternata*, thus leading to a general belief that it is the most common species isolated in food products.

Chickpea is sown in Argentina between April and June during the autumn season; thus, part of the crop cycle coincides with a gradual decrease in average temperature and luminous intensity, a shortened photoperiod, and scarce rainfall. The rest of the cycle (mainly the reproductive phase) unfolds under a continuous increase in temperature, luminous intensity, and rainfall [38]. The optimum temperature for the chickpea’s reproductive phase ranges from 5 °C to 30 °C. For all these reasons, the temperatures used in the present study where chosen to simulate the conditions in which this crop grows in the fields. In addition, the a_W_ values used in the present study cover the fluctuation of this parameter during early seed formation (R5), until the seeds reach full physiological maturity (R8) [39]. On the other hand, these conditions of a_W_ and temperatures can also be present during inappropriate storage.

In the present study, not only temperature, but also a_W_ affected growth, and the pattern obtained was independent of the strains/species evaluated. Optimal a_W_ levels for growth were 0.99 to 0.95, with an optimal temperature ranging between 20 and 30 °C and the highest growth rates for the three strains at 0.99 a_W_ and 25 °C. We defined as optimal those growth rates that were above 50% of the maximum growth rates obtained. No growth was observed at ≤0.92 a_W_ at 4 °C. 

Previous studies about the requirements of water and temperature for *A. alternata* growth have revealed that this fungus requires a minimum of 0.84–0.88 a_W_, and optimal growth has been observe at 0.98 to 1.0 a_W_ at 25 °C [26,27,28,29,40,41].

The effect of environmental conditions on *A. arborescens* growth has not been previously studied in depth. Only few attempts can be found in the literature. Vaquera et al. [40] studied the effect of a_W_ and temperature on the growth of one strain of *A. arborescens* in a tomato-based media. They informed that the highest growth rate was observed at 0.995 a_W_ and 30 °C (7.21 mm/day), while the lowest occurred at 0.950 a_W_ and 6 °C, but they did not find statistical differences between the growth rates obtained at 0.975 a_W_ at 30 °C and 0.995 a_W_ at 25 and 30 °C. Camiletti et al. [42] studied the effect of temperature (10, 15, 20, 25, 30, and 35 °C) on mycelial growth of *A. arborescens* and *A. alternarta* strains associated with preharvest fruit drop in California citrus in Potato Dextrose Agar (with freely available water ≈ 0.995 a_W_). They observed that *A. alternata* had the capacity to grow faster than *A. arborescens* at all the evaluated temperatures, except at 25 and 35 °C, when the growth patterns were similar for both species. Our results for *A. arboresces* are in agreement with those obtained by Oviedo et al. [41] and partially agree with those informed by Camiletti et al. [42]. It is important to point out that, according to our results, *A. arboresces* grows significantly (*p* < 0.05) faster than both *A. alternata* strains, even though the growth patterns obtained through all the conditions were the same. 

In the present study, both variables (a_W_ and temperature) affected mycotoxin production, and the pattern obtained was dependent on the strains/species evaluated. In general, both *A. alternata* strains produced maximum amounts of AOH and AME at the highest temperature analyzed, 30 °C and 0.99–0.98 a_W_. For TA production, both *A. alternata* strains behaved completely different; RC-CR76 maximum levels were obtained at 25 °C and 0.96 a_W,_ and for RC-CR902, the conditions were 30 °C and 0.98 a_W_. For *A. arborescens*, maximum AOH, AME, and TA production occurred at the same conditions: 25 °C and 0.98 a_W_. In general, all these a_W_ and temperature conditions were not optimal for growth and were slightly narrower.

There are several studies in the literature dealing with the effect of a_W_ and temperature on mycotoxin production by *A. alternata* strains on natural substrates or on media-based natural substrates [26,27,28,29,41,43], but only a few have been performed with *A. arborescens* [40,44]. It is important to remark that none of them have been performed on chickpea. However, in general, the results of the present work are similar to those obtained by the authors mentioned above. Although the basic culture media (chickpea, soybean, grape, tomato pulp, etc.) may vary, mycotoxin production tends to be similar under controlled environmental conditions. 

Because *Alternaria* section *Alternata* species (mainly *A. alternata*) are isolated with a high frequency in chickpea in Argentina and worldwide [8] and the natural occurrence of AOH and AME in this substrate has been informed, the contour maps developed with the results of the present study can provide very useful information to effectively facilitate risk prediction about growth and mycotoxin production during chickpea grain development, harvesting, and storage. All the reported information is valid because the a_W_ and temperature ranges used in these assays simulated the ranges that occur during chickpea development in the field and those that can be found during improper storage. 

## 5. Conclusions

So far, there is insufficient monitoring data on *Alternaria* mycotoxin occurrence and an absence of statutory or guideline limits set for these compounds in foods and feeds by regulatory authorities worldwide. However, there is increasing awareness among European authorities and the scientific community about the high incidence rate of *Alternaria* toxins in relevant foodstuffs and about population exposure through diet [45]. It is well known that the presence of *Alternaria* toxins in food can be reduced or prevented by using good agricultural practices, good storage and transport conditions, and good manufacturing practices. The present study provides useful information in that direction.

## Figures and Tables

**Table 1:** Analysis of variance on the effects of strain (S), temperature (T), and water activity (a_W_) and their interactions on growth rate by three strains of *Alternaria* section *Alternaria* in chickpea-based media.

Source of Variation	df ^a^	Growth Rate (mm/day)	
		MS ^b^	F ^c^
S	2	0.0285	12.70 *
T	3	131.071	57,309.634 *
a_W_	6	84.340	36,876.853 *
S × T	6	0.00757	3.311 *
S × a_W_	12	0.103	45.225 *
T × a_W_	18	9.292	4063.036 *
S × T × a_W_	36	0.0287	12.530 *

* Significant *p* ≤ 0.001; ^a^ Degrees of freedom; ^b^ Mean square; ^c^ Snedecor-F.

**Table 2:** Analysis of variance on the effects of water activity (a_W_), temperature (T), and incubation time (D) and their interactions on alternariol (AOH), alternariol monomethyl ether (AME), and tenuazonic acid (TA) production by two *Alternaria alternata* strains: RC-CR767 and RC-CR902 and *Alternaria arborescens* RC-CR910 in chickpea-based media.

***Alternaria alternata* RC-CR767**							
**Source of Variation**	**df ^a^**	**AOH**		**AME**		**TA**	
		**MS ^b^**	**F ^c^**	**MS**	**F**	**MS**	**F**
T	3	52.75	2375.63 *	13.224	133.42 *	10.82	267.09 *
a_W_	6	7.56	340.66 *	2.727	42.13 *	6.67	167.70 *
D	3	10.03	451.66 *	2.460	62.44 *	8.91	219.82 *
T × a_W_	18	1.46	65.96 *	0.919	31.55 *	1.35	33.30 *
T × D	9	1.05	47.13 *	0.486	59.53 *	0.87	21.35 *
a_W_ × D	18	0.80	36.25 *	0.319	12.79 *	0.92	22.69 *
T × a_W_ × D	54	0.57	25.76 *	0.171	11.61 *	0.75	18.48 *
***Alternaria alternata* RC-CR902**							
**Source of Variation**	**df ^a^**	**AOH**		**AME**		**TA**	
		**MS ^b^**	**F ^c^**	**MS**	**F**	**MS**	**F**
T	3	61.38	923.72 *	19.80	1940.48 *	14.19	1370.11 *
a_W_	6	15.82	753.56 *	5.20	509.63 *	25.94	2503.82 *
D	3	11.07	527.21 *	2.1	204.44 *	8.58	828.23 *
T × a_W_	18	1.90	90.44 *	1.34	131.32 *	1.18	114.27 *
T × D	9	1.05	50.06 *	0.72	70.22 *	2.12	204.67 *
a_W_ × D	18	0.56	26.73 *	0.19	19.25 *	1.59	153.18 *
T × a_W_ × D	54	0.86	40.94 *	0.28	27.47 *	1.08	104.26 *
***Alternaria arborescens* RC-CR910**							
**Source of Variation**	**df ^a^**	**AOH**		**AME**		**TA**	
		**MS ^b^**	**F ^c^**	**MS**	**F**	**MS**	**F**
T	3	11.91	217.51 *	40.16	2062.23 *	7.42	460.94 *
a_W_	6	55.65	1016.64 *	8.16	419.46 *	3.69	229.42 *
D	3	13.20	241.16 *	6.470	332.22 *	6.24	387.69 *
T × a_W_	18	1.93	35.31 *	1.82	93.77 *	0.56	35.24 *
T × D	9	0.51	21.38 *	0.77	39.46 *	0.56	35.11 *
a_W_ × D	18	1.17	9.41 *	0.56	28.96 *	0.39	24.6 *
T × a_W_ × D	54	0.48	8.73 *	0.49	25.31 *	0.44	27.69 *

* Significant *p* ≤ 0.001; ^a^ Degrees of freedom; ^b^ Mean square; ^c^ Snedecor-F.

## Data Availability

Not applicable.
